# Establishing the validity and reliability of the Project Talent Personality Inventory

**DOI:** 10.3389/fpsyg.2013.00968

**Published:** 2013-12-24

**Authors:** Julie Pozzebon, Rodica I. Damian, Patrick L. Hill, Yuchen Lin, Susan Lapham, Brent W. Roberts

**Affiliations:** ^1^Department of Psychology, University of Illinois at Urbana-ChampaignChampaign, IL, USA; ^2^Department of Psychology, Carleton UniversityOttawa, ON, Canada; ^3^American Institutes for ResearchWashington, DC, USA

**Keywords:** Project Talent, personality scale, Project Talent Personality Inventory, test-retest reliability, construct validity

## Abstract

Project Talent is a national longitudinal study that started in 1960. The original sample included over 440,000 students, which amounted to a 5% representative sample of high school students across the United States. Previous research has not yet established the validity and reliability of the personality measure used in this study, that is, the Project Talent Personality Inventory (PTPI). Given the potential interest and use of the PTPI in forthcoming research, the goals of the present paper were to establish (a) the construct and predictive validity and (b) the internal consistency and test-retest reliability of the PTPI. This information will be valuable to researchers who might be interested in using the PTPI to predict life course outcomes, such as mortality, occupational success, relationship success, and health. Study 1 found that the 10 sub-scales of the PTPI showed good internal consistency reliability, as well as good construct and predictive validity. With the use of several modern personality measures, we showed how the 10 PTPI scales can be mapped onto the Big Five personality traits, and we examined their relations with health, well-being, and life satisfaction outcomes. Study 2 found that the 10 PTPI scales showed good test-retest reliability. Together, these findings allow researchers to better understand and use the PTPI scales, as they are available in Project Talent.

## Introduction

Project Talent is a national longitudinal study that started in 1960. The original sample included over 440,000 high school students from 1353 schools across the United States, which amounted to a 5% representative sample of high school students. The original assessment included measures of intelligence, interests, educational aspirations, high school experiences, attitudes, as well as an exhaustive list of background factors. As a result of the large sample and rich array of measures, the Project Talent data have been used to investigate a wide variety of topics, ranging from Vietnam War experiences (Card, 1983) to the study of cognitive abilities (e.g., Humphreys, 1988; Hedges and Nowell, 1995) and vocational interests (e.g., Steel et al., 1982; Tannen, 1983; Austin and Hanisch, 1990). However, one crucial measure that was included in the original 1960 assessment of Project Talent has been almost entirely overlooked: the personality trait measure (henceforth, the Project Talent Personality Inventory, or PTPI). With the Project Talent study garnering more attention now that the participants are passing through major milestones like the transition to retirement, there is renewed interest in the personality trait measure contained therein. Unfortunately, very little information is available on the development and validity of the PTPI.

Given the potential interest and use of the PTPI in forthcoming research, the goals of the present paper were to establish (a) the construct and predictive validity and (b) the internal consistency and test-retest reliability of the Project Talent Personality Inventory (PTPI). This information will be valuable to researchers who might be interested in using the PTPI to predict life course outcomes, such as mortality (Zhang et al., 2013), occupational success, relationship success, and health. Study 1 addresses the construct and predictive validity, as well as the internal consistency reliability of the PTPI. Study 2 addresses the test-retest reliability of the PTPI.

### The project talent personality inventory

The PTPI was designed to measure personality characteristics specific to a normal high-school student population. The test was created in order to investigate the link between personality in adolescence and post high school life success. Thus, during the PTPI item generation phase, items were created to meet two main criteria: they had to represent aspects of high school behavior and they had to be clear and identifiable to high school students (see Flanagan et al., 1960). Items were generated based on (a) a rational analysis (i.e., intuitive brainstorming of behaviors relevant to high-school students), (b) an adjectival analysis (i.e., a lexical approach looking at 2000 adjectives and 200 words from a thesaurus that were relevant to high school behavior), (c) an analysis of Allport and Odbert's (1936) exhaustive list of traits, and (d) the relevance of the selected items to broad personality traits previously identified in research at the time, such as, dominance, sociability, drive, self-sufficiency, masculinity, and maturity.

The above item generation approach led to a total of 300 items, forming 18 trait scales, which were tested on a preliminary sample of 736 high school students across four different schools. Following an item analysis, about half the items were eliminated because (a) they were not consistent with the rest of the items, (b) they recorded a high percentage of “omits,” (c) they showed inadequate item response distribution, or (d) the scales proved to be unreliable (Flanagan et al., 1960). Furthermore, several scales were excluded, and some scales were combined resulting in the final version of the inventory, which contained 150 items and 10 standard scales (see Appendix and Table A1). The 10 scales were labeled Vigor, Calmness, Mature Personality, Impulsiveness, Self-Confidence, Culture, Sociability, Leadership, Social Sensitivity, and Tidiness.

Although a validation study of the PTPI has never been published in a peer-reviewed outlet, initial attempts at construct validation showed that the PTPI related as expected to other personality trait measures popular at the time, including the California Psychological Inventory (Gough, 1957) and the Edwards Personal Preference Schedule (Edwards, 1953). Moreover, the PTPI also showed good predictive validity with respect to a variety of outcomes, including academic achievement, self-perceived health, participation in athletics, cultural activities, and organized social activities (Flanagan et al., 1964). For example, the Mature Personality scale predicted higher achievement and aptitude scores, as well as better health, participation in more social activities, and a higher intention to attend college. The Culture scale predicted greater participation in social, religious, and artistic activities. The Self-Confidence and Sociability scales predicted more involvement in social activities, and the Leadership scale predicted more involvement in organizational activities and higher intention to attend college.

Given the lack of awareness of the existence of the measure, the PTPI has been used in very few peer-reviewed articles. One study (Hynes et al., 1979) showed that, of the PTPI subscales, Self-Confidence, Mature Personality, Social Sensitivity, and Vigor predicted more leadership behavior, as indicated by objective biographical measures such as membership in various high school clubs, community organizations, and roles in these groups. Another study (Bonaccio and Reeve, 2006) showed that the PTPI Calmness subscale was highly (and negatively) correlated with the neuroticism scale from Goldberg and colleagues' International Personality Item Pool (IPIP; 2006). Finally, in the context of a paper investigating personality-intelligence links (Reeve et al., 2006), a small pilot study was conducted where the PTPI facets were factor analyzed together with the NEO scales, using the IPIP short form (Goldberg, 1999). The authors found preliminary evidence that the 10 PTPI facets loaded on the five factor model as follows: Social Sensitivity loaded with Agreeableness; Sociability, Leadership, Impulsiveness, Vigor, and Self-confidence loaded with Extraversion; Calmness loaded with Emotional stability; Tidiness and Maturity loaded with Conscientiousness; and Culture loaded with Openness.

The preliminary work on the PTPI provides some evidence for the validity of the scales, but not enough information for researchers to truly understand the meaning of each of the 10 primary scales available in the Project Talent database. In order to establish that type of information, we correlated the PTPI scales with a broader, more detailed set of personality trait scales, as well as a broad array of potential correlates, such as life satisfaction, well-being, and physical health.

## Study 1

Study 1 addressed the construct and predictive validity, as well as the internal consistency reliability of the PTPI. To investigate the PTPI's construct validity, we examined its relation to six other existing and well-established personality inventories including: (a) the Midlife Development in the U.S. Personality Scale (MIDUS; Rossi, 2001), (b) the International Personality Item Pool “version” of the Revised NEO Personality Inventory (NEO-PI-R; Costa and McCrae, 1992; IPIP-NEO; Goldberg et al., 2006), (c) the Chernyshenko Conscientiousness Scales (CCS; Hill and Roberts, 2011), (d) the Big Five Inventory (BFI; John et al., 1991), (e) the Short Grit Scale (Grit-S; Duckworth and Quinn, 2009), and (f) the Narcissistic Personality Inventory (NPI; Raskin and Terry, 1988). To investigate the PTPI's predictive validity, we examined its relation to measures of physical health and well-being—the Health Behavior Checklist (HBC; Vickers et al., 1990) and the SF-36 (Ware et al., 2000)—as well as its relation to life satisfaction, as measured by the Satisfaction with Life Scale (SWLS; Diener et al., 1985). We also investigated personality differences on the PTPI scales based on age and gender.

### Methods

#### Participants

Six thousand four hundred and thirty-two participants were recruited in the fall of 2011 with the aid of Zoomerang^1^, an online survey company that uses a nation-wide sampling frame. The 6432 participants (3934 after the data cleaning; see procedures below) were randomly assigned to three different survey sets; all three survey sets included the PTPI, but they differed in terms of the other personality and health measures assessed. This had to be done in order to keep survey duration at about 1 h to prevent participant fatigue. Thus, a subset of 2124 people (1235 after data cleaning) completed the PTPI, the IPIP-NEO, and the MIDUS; another subset of 2114 people (1431 after data cleaning) completed the PTPI, the CCS, the BFI, the Grit-S, the HBC, and the SF-36; the remaining 2194 (1268 after data cleaning) people completed the PTPI, the NPI, and the SWLS. A power analysis showed that the final sample sizes were appropriate, because to detect a small effect of 0.2 (which is typical of psychological research) with a power of 0.8, the required number of participants would be 193.

From the original 6432 participants, 2498 participants were removed prior to any analyses for the following reasons: (a) 422 of them answered “no” to the informed consent, (b) 1424 of them answered less than half of all the questions included in the specific survey subset they took part in, (c) 542 of them failed the integrity checks embedded in the survey, and (d) 110 of them finished the entire survey subset in less than 15 min, which was about two standard deviations below the mean duration. Such times suggest that the participants failed to fill in the surveys responsibly, particularly given that simply clicking through every question without reading a single word took the research team up to 12 min. Furthermore, if participants missed too many items from a particular scale (i.e., more than 1 in 8 items) that particular scale mean was not computed, and thus, not included in the analyses.

Of the remaining 3934 participants, there were 1374 men, 2484 women, and 76 unspecified. Participant ages ranged from 17 to 90 years (*M* = 49.98, *SD* = 19.32) and the sample included a variety of racial backgrounds (3455; Caucasian/European Americans, 211 African Americans, 112 Asian Americans, 58 Native Americans, 13 Pacific Islanders, and 85 unspecified). The sampling of participants in this dataset was largely focused on young (age 20s) and older (age 60s) groups in order to test the validity of the PTPI in samples close to the age of the PT sample when they took the test (i.e., 18) and the age the PT sample is now (i.e., 68).

All data obtained from the participants' responses to the surveys were encrypted to ensure data security. Through this system, participants were paid in Zoompoints that could be spent in the Zoomerang online system. The amount of points they received for this survey was roughly equivalent to 2 USD. All the measures, syntax, and de-identified data used in this paper can be found at the following link: https://openscienceframework.org/project/aTJXc/

#### Measures

***Demographics***. Participants responded to questions about their age, gender, ethnicity, marital status, education level, income, and political and religious affiliation.

***Integrity checks***. Several integrity check items were included randomly in the survey subsets to ensure that participants were paying attention to and understanding the questions. These items asked participants to choose a specific response (e.g., *Answer number 3 to this item*).

***Project Talent Personality Inventory***. Participants completed 150 PTPI items, from which the 10 PTPI scales were scored. The original version of the PTPI included 150 items and 13 scales, but only 10 of these scales (including 108 items) were actually scored in Project Talent, and are thus useful to researchers^2^. These are the items and scales that we focused on here. For each item, participants rated how well the item described them on a 5-point scale ranging from 1 (*Not very well)* to 5 (*Extremely well*). The Vigor scale measures the physical activity level of a person (e.g., *I play games for hours without getting tired*). The Calmness scale measures the ability to react to emotional situations in an appropriate manner without displaying extreme emotions (e.g., *I rarely lose my temper*). The Mature Personality scale measures the ability to get work done efficiently, to work on a project to completion, and to accept assigned responsibility (e.g., *I work fast and get a lot done, people say they can count on me*). The Impulsiveness scale measures the tendency to make quick decisions without full consideration of the outcomes (e.g., *I usually act on the first plan that comes to mind*). The Self-Confidence scale measures one's feelings of social acceptability and the willingness to act and think independently (e.g., *I'm equal to any occasion*). The Culture scale measures the tendency to recognize the value of aesthetic things, and to display refinement and good taste (e.g., *I enjoy works of art*). The Sociability scale measures the tendency to enjoy being with people as well as to be optimistic (e.g., *I take a big part in social activities, I am good natured most of the time*). The Leadership scale measures activities such as taking charge and seeking out responsibilities (e.g., *I like to make decisions*). The Social Sensitivity scale measures the propensity to put oneself in another's place (e.g., *I don't like to see someone's feelings hurt*). Finally, the Tidiness scale measures the desire for order and neatness in one's environment (e.g., *I do my homework as neatly as possible*). To score the 10 scales, we used continuous 5-point Likert scores and averaged them across the appropriate items, reverse scoring items where necessary, as indicated in Table A1 of the Appendix. Scale reliabilities and descriptive statistics are presented in Tables 1 and 2. All the analyses presented in the paper use the continuous 1 (*not very well*) to 5 (*extremely well*) Likert scale coding. Even though the 1960 original items used in Project Talent included the 5 answer choices with the same anchors, the answer choices were labeled A, B, C, D, and E (as opposed to 5–1), and they were coded in a dichotomous manner prior to the scale construction. The original dichotomous coding was as follows: answers A (*extremely well*) and B (*quite well*) were coded as 1, whereas answers C (*fairly well*), D (*slightly*), and E (*not very well*) were coded as 0; in the case of reverse scored items, answers D and E were coded as 1, whereas answers A, B, and C were coded as 0. We have also conducted reliability analyses using the original dichotomous coding and report the results where appropriate.

**Table 1:** **Reliabilities, means, and standard deviations in the Project Talent Personality Inventory Scales broken down by age**.

**Scale**	**Total**			**20s**			**60s**			***d* (20–60)**
	**α**	***M***	***SD***	**α**	***M***	***SD***	**α**	***M***	***SD***	
Vigor	0.89	2.93	0.91	0.86	3.04	0.83	0.90	2.86	0.94	0.18
Calmness	0.88	3.90	0.69	0.87	3.71	0.72	0.88	4.01	0.65	−0.42
Mature personality	0.93	3.92	0.60	0.93	3.78	0.63	0.92	4.01	0.56	−0.38
Impulsiveness	0.67	2.50	0.55	0.72	2.50	0.61	0.65	2.49	0.52	0.02
Self confidence	0.81	3.42	0.66	0.78	3.12	0.64	0.79	3.59	0.61	−0.71
Culture	0.84	3.54	0.66	0.81	3.52	0.62	0.86	3.55	0.68	−0.06
Sociability	0.84	3.16	0.68	0.83	3.10	0.68	0.85	3.19	0.67	−0.13
Leadership	0.82	2.65	0.85	0.79	2.64	0.82	0.84	2.65	0.86	−0.01
Social sensitivity	0.85	3.89	0.65	0.85	3.85	0.67	0.85	3.93	0.63	−0.14
Tidiness	0.88	3.57	0.73	0.86	3.50	0.72	0.89	3.61	0.74	−0.15

Ns = 3907–3926 for total sample, N for the 20s = 1243–1246, N for the 60s = 2405–2420. The remaining participants were not in their 20s or 60s. Minor differences in Ns across scales were due to different numbers of missing items on each scale, which sometimes prevented scale computation, as indicated in the Data Cleaning section.

**Table 2:** **Reliabilities, means, and standard deviations in the Project Talent Personality Inventory Scales broken down by gender**.

**Scale**	**Men**			**Women**			***d* (Men-Women)**
	**α**	***M***	***SD***	**α**	***M***	***SD***	
Vigor	0.89	3.03	0.89	0.88	2.87	0.91	0.17
Calmness	0.88	3.90	0.66	0.88	3.90	0.71	0.00
Mature personality	0.93	3.90	0.58	0.93	3.93	0.61	−0.05
Impulsiveness	0.65	2.57	0.52	0.68	2.46	0.57	0.18
Self confidence	0.78	3.60	0.60	0.81	3.32	0.67	0.41
Culture	0.85	3.39	0.67	0.83	3.61	0.64	−0.32
Sociability	0.85	3.18	0.67	0.84	3.15	0.68	0.06
Leadership	0.82	2.81	0.82	0.82	2.56	0.84	0.29
Social sensitivity	0.83	3.71	0.63	0.85	3.99	0.64	−0.42
Tidiness	0.88	3.49	0.71	0.88	3.61	0.74	−0.17

Ns for men = 1360–1372, Ns for women = 2467–2479. The remaining 76 participants did not indicate their gender.

***Midlife Development in the U.S. Personality Scale (MIDUS; Rossi, 2001***). Participants completed the MIDUS scale consisting of 25 single-word items designed to measure the Big Five personality dimensions: Agreeableness (e.g., *warm*), Neuroticism (e.g., *worrying*), Conscientiousness (e.g., *responsible*), Extraversion (e.g., *outgoing*), and Openness (e.g., *creative*). Participants were asked to rate how descriptive each of the items was of themselves on a 4-point scale ranging from 1 (*Not at all*) to 4 (*A lot)*. In the present sample, the five subscales of the survey showed good internal consistency reliabilities ranging from 0.68 (Conscientiousness) to 0.85 (Agreeableness).

***International Personality Item Pool NEO scales (Goldberg et al., 2006)***. The IPIP-NEO scales contain 300 items designed to measure the Big Five personality dimensions similarly to the NEO-PI-R. Participants rated how accurately each item described them on a 5-point scale ranging from 1 (*Very inaccurate*) to 5 (*Very accurate)*. The 30 facet scales (Neuroticism: anxiety, anger, depression, self-consciousness, immoderation, vulnerability; Extraversion: friendliness, gregariousness, assertiveness, activity level, excitement seeking, cheerfulness; Openness: imagination, artistic interests, emotionality, adventurousness, intellect, liberalism; Agreeableness: trust, morality, altruism, cooperation, modesty, sympathy; Conscientiousness: self-efficacy, orderliness, dutifulness, achievement striving, self-discipline, and cautiousness) all had good internal consistency reliabilities with the alphas ranging from 0.61 for activity level (Extraversion) to 0.90 for depression (Neuroticism). At the factor level, internal consistency reliabilities were all equal to or above 0.90.

***Chernyshenko Conscientiousness Scales (CCS; Hill and Roberts, 2011)***. The CCS contains 60 items measuring six facets of Conscientiousness: Order (e.g., *I become annoyed when things around me are disorganized*), Industriousness (e.g., *I make every effort to do more than what is expected of me*), Self-Control (e.g., *I rarely jump into something without first thinking about it*), Traditionalism (e.g., *I support long-established rules and traditions*), Responsibility (e.g., *I carry out my obligations to the best of my ability*), and Virtue (e.g., *I would lie without hesitation if it serves my purpose*, reverse scored). Participants were asked to rate their agreement with each of the items on a 4-point scale ranging from 1 (*Disagree strongly*) to 4 (*Agree strongly*). The internal consistency reliabilities of these scales were all good with alphas exceeding 0.74.

***Big Five Inventory (BFI; John et al., 1991)***. The BFI consists of 44 items designed to measure the Big Five dimensions of personality: Agreeableness (e.g., *I see myself as someone who is considerate and kind to almost everyone*), Neuroticism (e.g., *I see myself as someone who is emotionally stable, not easily upset*, reverse scored), Conscientiousness (e.g., *I see myself as someone who is a reliable worker*), Extraversion (e.g., *I see myself as someone who is outgoing, sociable*), and Openness (e.g., *I see myself as someone who is curious about many different things*). Participants were asked to rate how well each of the characteristics applied to them on a 5-point scale ranging from 1 (*Disagree strongly*) to 5 (*Agree strongly*). The scales showed good internal consistency reliability with alphas exceeding 0.82.

***Short Grit Scale (Grit-S; Duckworth and Quinn, 2009)***. The Grit-S consists of eight items designed to measure people's grit, defined as a passion for long-term goals, coupled with perseverance and a powerful motivation to overcome obstacles and achieve the respective objectives. Thus, Grit-S has two facets, namely, interest in long-term goals (e.g., *I have difficulty maintaining my focus on projects that take more than a few months to complete*, reverse scored) and perseverance in the face of obstacles (e.g., *Setbacks don't discourage me*). Participants were asked to rate how well each of the descriptions applied to them on a 5-point scale ranging from 1 (*Not at all like me*) to 5 (*Very much like me*). The scales showed good internal consistency reliability, as follows: interest (0.80), perseverance (0.70), and overall grit (0.80).

***Narcissistic Personality Inventory (NPI; Raskin and Terry, 1988)***. The NPI consists of 40 pairs of opposing statements (e.g., *I like having authority over people* vs. *I don't mind following orders*). For each of these pairs, participants had to make a forced choice by identifying the one item that represented them best. The items can be grouped into seven different facets of narcissism: Authority (e.g., *I have a natural talent for influencing people*), Self-sufficiency (e.g., *I like to take responsibility for making decisions*), Superiority (e.g., *I think I am a special person*), Exhibitionism (e.g., *I will usually show off if I get the chance*), Exploitativeness (e.g., *I can make anybody believe anything I want them to*), Vanity (e.g., *I like to look at my body*), and Entitlement (e.g., *I will never be satisfied until I get all that I deserve*). Internal consistency reliabilities for the different facets ranged from 0.49 (self-sufficiency and entitlement) to 0.81 (authority), and the overall reliability of the NPI was 0.88.

***Health Behavior Checklist (HBC; Vickers et al., 1990)***. The HBC consists of 40 items designed to measure a variety of health behaviors that define four scales: wellness maintenance (e.g., *I exercise to stay healthy*), accident control (e.g., *I have a first aid kit in my home*), traffic risk (e.g., *I carefully obey traffic rules so I won't have accidents*), and substance use risk (e.g., *I don't smoke*). Participants indicated how much they agreed with each item on a 5-point scale ranging from 1 (*Disagree strongly*) to 5 (*Agree strongly*). Internal consistency reliabilities for these scales were 0.46 for substance use risk, and above 0.73 for the other three measures.

***SF-36 Ware et al., 2000***. The SF-36 is a 36-item inventory measuring eight scales of functional health and well-being: physical functioning, role limitations due to physical health, role limitations due to emotional problems, energy, emotional well-being, social functioning, bodily pain, and general health. The items employed various scales, ranging from dichotomous scales of involvement in various health behaviors, to 5-point Likert scales (*Strongly disagree* to *Strongly agree*), where participants indicated their level of agreement with subjective health assessments. Because the item format differed substantially within and across scales, z-scores were computed for all the items before computing scale scores. Internal consistency reliabilities for the scales were all above 0.79 in this sample.

***Satisfaction with Life Scale (SWLS; Diener et al., 1985)***. The SWLS consists of five items measuring life satisfaction (e.g., *In most ways my life is close to ideal*). Participants rated their agreement with each of the items on a 7-point scale ranging from 1 (*Strongly Disagree*) to 7 (*Strongly Agree*). The internal consistency reliability of this scale was 0.91.

### Results

#### PTPI: descriptive statistics, internal consistency reliabilities, and sub-scale correlations

Internal consistency reliabilities and descriptive statistics for the PTPI scales are reported in Table 1 for the total sample, the 20s age group, and the 60s age group. The reliabilities were generally good with most scale alphas exceeding 0.81, the only exception being Impulsiveness, which had a reliability of 0.67. When using the original dichotomous coding of the PTPI items in Study 1 (see Measures section), internal consistency reliabilities were: Vigor (0.82), Calmness (0.85), Mature Personality (0.91), Impulsiveness (0.56), Self-Confidence (0.77), Culture (0.79), Sociability (0.79), Leadership (0.76), Social Sensitivity (0.82), Tidiness (0.85). Thus, the average decrement in alpha was 0.05.

As shown in Table 1, the means for all the scales were reasonably close to the theoretical midpoints (i.e., 3.00) and the standard deviations were reasonably wide. There was no evidence of significantly skewed or kurtotic data.

The correlations among the 10 PTPI scales are shown in Table 3. The correlations among the scales were mostly positive with the exception of Impulsiveness, which was negatively correlated with five of the scales (Calmness, Mature Personality, Culture, Social Sensitivity, and Tidiness). The scales were generally highly correlated with one another with most correlations exceeding 0.30, and the following correlations were above 0.60: Calmness correlated 0.66 with Mature Personality and 0.61 with Social Sensitivity; Mature Personality correlated 0.63 with Social Sensitivity and 0.62 with Tidiness; and Culture correlated 0.65 with Social Sensitivity.

**Table 3:** **Intercorrelations among the Project Talent Personality Inventory scales**.

**Scale**	**1**	**2**	**3**	**4**	**5**	**6**	**7**	**8**	**9**	**10**
Vigor		0.32	0.48	0.18	0.31	0.43	0.45	0.50	0.28	0.39
Calmness			0.66	−0.16	0.49	0.54	0.36	0.30	0.61	0.47
Mature personality				−0.09	0.45	0.58	0.39	0.46	0.63	0.62
Impulsiveness					0.07	−0.05	0.17	0.28	−0.14	−0.13
Self-confidence						0.23	0.39	0.41	0.18	0.21
Culture							0.46	0.40	0.65	0.57
Sociability								0.43	0.44	0.30
Leadership									0.27	0.28
Social sensitivity										0.45
Tidiness										

Ns = 3899–3918. All values are significant at p <0.01. Values show correlations for the total sample.

#### Construct validity

The correlations between the PTPI and previously established personality scales are shown in Table 4. The 10 PTPI scales were correlated with the MIDUS personality scale, the IPIP-NEO facets and factors, the CCS, the BFI, the Grit-S, and the NPI. High correlations (above 0.50) are noted here, and they are organized by PTPI scale.

**Table 4:** **Correlations between the PTPI scales and Established Personality Scales**.

**Scale**	**Vigor**	**Calmness**	**Mature personality**	**Impulsiveness**	**Self-confidence**	**Culture**	**Sociability**	**Leadership**	**Social sensitivity**	**Tidiness**
**MIDUS B5**										
Neuroticism	−0.24^*^	−0.55^*^	−0.37^*^	−0.05	−0.69^*^	−0.22^*^	−0.25^*^	−0.26^*^	−0.21^*^	−0.19^*^
Extraversion	0.56^*^	0.31^*^	0.41^*^	0.22^*^	0.44^*^	0.40^*^	0.69^*^	0.47^*^	0.35^*^	0.31^*^
Openness	0.42^*^	0.28^*^	0.36^*^	0.14^*^	0.26^*^	0.50^*^	0.33^*^	0.43^*^	0.35^*^	0.25^*^
Agreeableness	0.20^*^	0.42^*^	0.42^*^	−0.04	0.12^*^	0.48^*^	0.44^*^	0.20^*^	0.66^*^	0.29^*^
Conscientiousness	0.31^*^	0.47^*^	0.68^*^	−0.22^*^	0.31^*^	0.40^*^	0.21^*^	0.20^*^	0.43^*^	0.62^*^
**IPIP**										
**Neuroticism**	−0.35^*^	−0.66^*^	−0.54^*^	−0.04	−0.81^*^	−0.33^*^	−0.35^*^	−0.40^*^	−0.33^*^	−0.31^*^
nAnxiety	−0.30^*^	−0.54^*^	−0.38^*^	−0.18^*^	−0.74^*^	−0.22^*^	−0.31^*^	−0.34^*^	−0.20^*^	−0.20^*^
nAnger	−0.23^*^	−0.71^*^	−0.41^*^	0.06	−0.56^*^	−0.30^*^	−0.28^*^	−0.22^*^	−0.38^*^	−0.24^*^
nDepression	−0.31^*^	−0.56^*^	−0.50^*^	−0.03	−0.72^*^	−0.28^*^	−0.32^*^	−0.32^*^	−0.29^*^	−0.26^*^
nSelf-consciousness	−0.29^*^	−0.37^*^	−0.42^*^	−0.19^*^	−0.79^*^	−0.25^*^	−0.41^*^	−0.46^*^	−0.18^*^	−0.20^*^
nImmoderation	−0.27^*^	−0.38^*^	−0.34^*^	0.23^*^	−0.34^*^	−0.21^*^	−0.08^*^	−0.14^*^	−0.18^*^	−0.31^*^
nVulnerability	−0.32^*^	−0.63^*^	−0.58^*^	−0.05	−0.75^*^	−0.33^*^	−0.30^*^	−0.42^*^	−0.33^*^	−0.29^*^
**Extraversion**	0.56^*^	0.35^*^	0.49^*^	0.30^*^	0.53^*^	0.43^*^	0.75^*^	0.60^*^	0.37^*^	0.26^*^
eFriendliness	0.34^*^	0.48^*^	0.48^*^	0.10^*^	0.61^*^	0.43^*^	0.74^*^	0.40^*^	0.48^*^	0.28^*^
eGregariousness	0.37^*^	0.22^*^	0.26^*^	0.22^*^	0.38^*^	0.30^*^	0.76^*^	0.37^*^	0.23^*^	0.17^*^
eAssertiveness	0.38^*^	0.23^*^	0.43^*^	0.23^*^	0.56^*^	0.30^*^	0.47^*^	0.71^*^	0.21^*^	0.18^*^
eActivity Level	0.51^*^	0.16^*^	0.46^*^	0.15^*^	0.24^*^	0.22^*^	0.25^*^	0.41^*^	0.15^*^	0.25^*^
eExcitement Seeking	0.38^*^	−0.09^*^	0.01	0.40^*^	0.02	0.12^*^	0.33^*^	0.27^*^	−0.01	0.00
eCheerfulness	0.39^*^	0.44^*^	0.43^*^	0.15^*^	0.37^*^	0.43^*^	0.51^*^	0.37^*^	0.45^*^	0.24^*^
**Openness**	0.16^*^	0.19^*^	0.28^*^	0.03	0.13^*^	0.42^*^	0.16^*^	0.22^*^	0.38^*^	0.03
oImagination	0.08^*^	0.01	0.06	0.09^*^	−0.05	0.20^*^	0.03	0.14^*^	0.17^*^	−0.08^*^
oArtistic Interests	0.15^*^	0.28^*^	0.32^*^	−0.09^*^	0.14^*^	0.50^*^	0.19^*^	0.11^*^	0.43^*^	0.18^*^
oEmotionality	0.03	0.05	0.22^*^	−0.09^*^	−0.16^*^	0.30^*^	0.14^*^	0.08^*^	0.41^*^	0.09^*^
oAdventurousness	0.28^*^	0.25^*^	0.31^*^	0.17^*^	0.39^*^	0.33^*^	0.34^*^	0.26^*^	0.26^*^	0.07^*^
oIntellect	0.21^*^	0.31^*^	0.41^*^	0.00	0.33^*^	0.37^*^	0.12^*^	0.39^*^	0.33^*^	0.10^*^
oLiberalism	−0.09^*^	−0.15^*^	−0.18^*^	0.04	−0.14^*^	−0.03	−0.12	−0.08^*^	−0.06^*^	−0.21^*^
**Agreeableness**	−0.01	0.50^*^	0.43^*^	−0.23^*^	0.24^*^	0.34^*^	0.24^*^	−0.05	0.58^*^	0.22^*^
aTrust	0.23^*^	0.45^*^	0.38^*^	0.07	0.41^*^	0.33^*^	0.43^*^	0.23^*^	0.40^*^	0.19^*^
aMorality	−0.11^*^	0.34^*^	0.36^*^	−0.34^*^	0.21^*^	0.16^*^	0.00	−0.17^*^	0.36^*^	0.20^*^
aAltruism	0.18^*^	0.55^*^	0.58^*^	−0.11^*^	0.34^*^	0.49^*^	0.42^*^	0.21^*^	0.72^*^	0.29^*^
aCooperation	−0.04	0.48^*^	0.30^*^	−0.27^*^	0.17^*^	0.27^*^	0.14^*^	−0.14^*^	0.46^*^	0.19^*^
aModesty	−0.27^*^	0.00	−0.06	−0.23^*^	−0.23^*^	−0.12^*^	−0.23^*^	−0.45^*^	0.05	−0.02
aSympathy	0.02	0.31^*^	0.27^*^	−0.14^*^	0.10^*^	0.32^*^	0.22^*^	0.04	0.50^*^	0.07
**Conscientiousness**	0.31^*^	0.59^*^	0.78^*^	−0.26^*^	0.46^*^	0.43^*^	0.23^*^	0.29^*^	0.51^*^	0.59^*^
cSelf-Efficacy	0.36^*^	0.60^*^	0.75^*^	−0.07	0.56^*^	0.47^*^	0.32^*^	0.45^*^	0.53^*^	0.41^*^
cOrderliness	0.20^*^	0.28^*^	0.44^*^	−0.20^*^	0.14^*^	0.28^*^	0.10^*^	0.09^*^	0.25^*^	0.74^*^
cDutifulness	0.09^*^	0.53^*^	0.60^*^	−0.29^*^	0.33^*^	0.32^*^	0.13^*^	0.06	0.49^*^	0.36^*^
cAchievement striving	0.43^*^	0.52^*^	0.81^*^	−0.04	0.43^*^	0.47^*^	0.33^*^	0.44^*^	0.51^*^	0.46^*^
cSelf Discipline	0.38^*^	0.48^*^	0.72^*^	−0.06	0.49^*^	0.35^*^	0.28^*^	0.34^*^	0.37^*^	0.54^*^
cCautiousness	−0.05	0.36^*^	0.32^*^	−0.61^*^	0.20^*^	0.15^*^	−0.09^*^	−0.06	0.24^*^	0.24^*^
**MIDUS-IPIP AVERAGE**										
Neuroticism	−0.31^*^	−0.64^*^	−0.48^*^	−0.05	−0.79^*^	−0.29^*^	−0.32^*^	−0.35^*^	−0.28^*^	−0.26^*^
Extraversion	0.60^*^	0.35^*^	0.48^*^	0.28^*^	0.52^*^	0.44^*^	0.77^*^	0.57^*^	0.39^*^	0.30^*^
Openness	0.33^*^	0.26^*^	0.36^*^	0.10^*^	0.22^*^	0.52^*^	0.29^*^	0.37^*^	0.41^*^	0.15^*^
Agreeableness	0.12^*^	0.52^*^	0.48^*^	−0.15^*^	0.20^*^	0.46^*^	0.39^*^	0.09^*^	0.71^*^	0.29^*^
Conscientiousness	0.33^*^	0.56^*^	0.78^*^	−0.26^*^	0.41^*^	0.45^*^	0.24^*^	0.26^*^	0.50^*^	0.65^*^
**CCS**										
Order	0.22^*^	0.27^*^	0.44^*^	−0.16^*^	0.17^*^	0.34^*^	0.13^*^	0.18^*^	0.25^*^	0.75^*^
Virtue	0.08^*^	0.41^*^	0.41^*^	−0.19^*^	0.29^*^	0.28^*^	0.17^*^	0.09^*^	0.37^*^	0.22^*^
Traditionalism	0.07	0.30^*^	0.31^*^	−0.21^*^	0.13^*^	0.23^*^	0.17^*^	0.05	0.27^*^	0.33^*^
Self-control	−0.06	0.44^*^	0.38^*^	−0.56^*^	0.22^*^	0.25^*^	−0.06	−0.04	0.35^*^	0.34^*^
Responsibility	0.23^*^	0.52^*^	0.68^*^	−0.18^*^	0.43^*^	0.39^*^	0.27^*^	0.26^*^	0.52^*^	0.44^*^
Industriousness	0.32^*^	0.49^*^	0.74^*^	−0.16^*^	0.40^*^	0.47^*^	0.29^*^	0.32^*^	0.52^*^	0.50^*^
Total conscientiousness	0.20^*^	0.55^*^	0.68^*^	−0.32^*^	0.37^*^	0.45^*^	0.23^*^	0.20^*^	0.51^*^	0.61^*^
**BFI**										
Neuroticism	−0.28^*^	−0.61^*^	−0.42^*^	−0.03	−0.73^*^	−0.27^*^	−0.32^*^	−0.29^*^	−0.25^*^	−0.24^*^
Extraversion	0.50^*^	0.21^*^	0.35^*^	0.27^*^	0.55^*^	0.30^*^	0.70^*^	0.48^*^	0.24^*^	0.23^*^
Openness	0.27^*^	0.23^*^	0.37^*^	0.06	0.24^*^	0.47^*^	0.22^*^	0.41^*^	0.34^*^	0.17^*^
Agreeableness	0.18^*^	0.61^*^	0.51^*^	−0.16^*^	0.38^*^	0.39^*^	0.39^*^	0.11^*^	0.65^*^	0.32^*^
Conscientiousness	0.28^*^	0.58^*^	0.77^*^	−0.22^*^	0.48^*^	0.43^*^	0.26^*^	0.27^*^	0.48^*^	0.66^*^
**Grit-S**										
Interest	0.16^*^	0.41^*^	0.47^*^	−0.22^*^	0.45^*^	0.18^*^	0.15^*^	0.10^*^	0.25^*^	0.38^*^
Perseverance	0.37^*^	0.49^*^	0.70^*^	−0.10^*^	0.42^*^	0.38^*^	0.27^*^	0.32^*^	0.40^*^	0.46^*^
Total Grit	0.30^*^	0.53^*^	0.67^*^	−0.19^*^	0.51^*^	0.32^*^	0.24^*^	0.23^*^	0.37^*^	0.49^*^
**NPI**										
Authority	0.34^*^	0.14^*^	0.33^*^	0.19^*^	0.41^*^	0.24^*^	0.33^*^	0.71^*^	0.12^*^	0.17^*^
Self-sufficient	0.32^*^	0.15^*^	0.26^*^	0.09^*^	0.23^*^	0.16^*^	0.11^*^	0.34^*^	0.05	0.20^*^
Superiority	0.25^*^	0.09^*^	0.14^*^	0.20^*^	0.20^*^	0.27^*^	0.28^*^	0.39^*^	0.11^*^	0.11^*^
Exhibitionism	0.17^*^	−0.11^*^	0.00	0.27^*^	0.13^*^	0.08^*^	0.26^*^	0.34^*^	−0.03	−0.02
Exploitative	0.23^*^	0.04	0.13^*^	0.21^*^	0.17^*^	0.16^*^	0.18^*^	0.43^*^	0.08^*^	0.09^*^
Vanity	0.20^*^	−0.01	−0.01	0.09^*^	−0.01	0.12^*^	0.10^*^	0.15^*^	0.04	0.05
Entitlement	0.17^*^	−0.08^*^	0.03	0.13^*^	0.01	0.12^*^	0.08^*^	0.31^*^	−0.07	0.11^*^
Total narcissism	0.36^*^	0.07	0.23^*^	0.25^*^	0.29^*^	0.25^*^	0.30^*^	0.61^*^	0.08^*^	0.16^*^

N for the relations with the MIDUS and IPIP (including facets) = 1182–1234. N for the relations with BFI, CCS, and Grit-S = 1340–1424. N for the relation with the NPI = 1192–1246

* p < 0.01.

The “MIDUS-IPIP Average” scores were computed by averaging the standardized domain scores across the two instruments in an effort to reduce unreliability and provide “cleaner” correlations with the PTPI scales (the BFI could not be included in the average because it was completed by a separate subset of participants).

*Vigor* correlated highly with the Extraversion scales across the MIDUS, IPIP-NEO (particularly with the Activity Level facet), and BFI.

*Calmness* correlated highly and negatively with the Neuroticism scales across the MIDUS, IPIP-NEO (particularly with the Anxiety, Anger, Depression, and Vulnerability facets), and BFI. Calmness also correlated highly and positively with the Agreeableness scale across the IPIP-NEO (particularly with the Altruism facet) and the BFI, and with the Conscientiousness scale across the IPIP-NEO (particularly with the Self-efficacy, Dutifulness, and Achievement striving facets), CCS (the total score and the Responsibility facet), and the BFI. Calmness also correlated highly with overall grit.

*Mature Personality* correlated highly with the Conscientiousness scales across the MIDUS, IPIP-NEO (particularly with the Self-efficacy, Dutifulness, Achievement striving, and Self-discipline facets), the CCS (the total score and the Responsibility and Industriousness facets), and the BFI. Mature personality also correlated highly and positively with the Agreeableness scales across the IPIP-NEO (particularly with the Altruism facet) and the BFI, and negatively with the Neuroticism scale of the IPIP-NEO (particularly with the Depression and Vulnerability facets). Mature personality was also related to more perseverance and total grit.

*Impulsiveness* correlated highly and negatively with the Cautiousness facet of Conscientiousness in the IPIP-NEO and with the Self-control facet of Conscientiousness in the CCS.

*Self-Confidence* correlated highly and negatively with the Neuroticism scales across the MIDUS, IPIP-NEO (particularly with the Anxiety, Anger, Depression, Self-consciousness, and Vulnerability facets), and BFI. Self-confidence also correlated positively with the Extraversion scales of the IPIP-NEO (particularly with the Friendliness and Assertiveness facets) and the BFI, as well as with the IPIP-NEO Conscientiousness scale (particularly the Self-efficacy facet), and the total grit score.

*Culture* correlated highly with the Openness scale in MIDUS and with the Artistic Interest facet of Openness in the IPIP-NEO.

*Sociability* correlated highly with the Extraversion scales across the MIDUS, IPIP-NEO (particularly with the Friendliness, Gregariousness, and Cheerfulness facets), and BFI.

*Leadership* correlated highly with the Extraversion scale of the IPIP-NEO (particularly the Assertiveness facet) and with Narcissism on the NPI (particularly the Authority facet).

*Social Sensitivity* correlated highly with the Agreeableness scales across the MIDUS, IPIP-NEO (particularly with the Altruism and Sympathy facets), and BFI. Social sensitivity also correlated highly with the Conscientiousness scales across the IPIP-NEO (particularly with the Self-efficacy and Achievement striving facets) and the CCS (the total score and the Responsibility and Industriousness facets).

*Tidiness* correlated highly with the Conscientiousness scales across the MIDUS, IPIP-NEO (particularly with the Orderliness and Self-discipline facets), the CCS (the total score and the Order and Industriousness facets), and the BFI.

#### Gender and age differences in PTPI scales

When the means were broken down by gender (see Table 2), moderate gender differences in the expected directions (see Schmitt et al., 2008) were found with women scoring higher on Culture, Social Sensitivity, and Tidiness. Men scored higher in Vigor, Impulsiveness, Self-Confidence, and Leadership. Likewise, age differences in the PTPI scales were consistent with previously published differences in the Big Five personality traits (see Roberts et al., 2006). Thus, the 60s age group (compared to the 20s age group) was higher in Calmness, Mature Personality, Self-Confidence, Sociability, Social Sensitivity, and Tidiness, but was lower in Vigor.

Some researchers may be interested in combining the PTPI scales to maximize their representation of each of the Big Five domains. To inform this possibility we conducted 15 separate multivariate models, where we regressed each of the higher-order traits of the three Big Five inventories (MIDUS, IPIP-NEO, and BFI) on all the 10 PTPI traits. In Table 5, we report how much of the variance in each of the Big Five traits could be accounted for with all the 10 PTPI traits included in a single model, and we also report standardized regression weights for each PTPI trait.

**Table 5:** **Multivariate regressions of each Big Five personality scale on the 10 PTPI Scales (included in the same model)**.

**Scale**	***R*^2^**	**Vigor**	**Calm-ness**	**Mature personality**	**Impulsive-ness**	**Self-confidence**	**Culture**	**Sociability**	**Leadership**	**Social sensitivity**	**Tidiness**
**MIDUS B5**											
Neuroticism	0.55^*^	−0.04	−0.39^*^	0.06	−0.08^*^	−0.57^*^	0.01	0.03	0.09^*^	0.05	0.02
Extraversion	0.58^*^	0.27^*^	−0.13^*^	−0.04	0.06^*^	0.24^*^	−0.03	0.45^*^	0.03	0.13^*^	0.06
Openness	0.34^*^	0.17^*^	−0.10^*^	−0.01	0.04	0.10^*^	0.41^*^	−0.02	0.15^*^	0.08	−0.10^*^
Agreeableness	0.47^*^	−0.06	0.00	0.02	0.02	−0.02	0.03	0.21^*^	−0.04	0.59^*^	−0.03
Conscientiousness	0.56^*^	0.01	−0.05	0.52^*^	−0.13^*^	0.10^*^	−0.09^*^	−0.04	−0.07^*^	0.03	0.36^*^
**IPIP**											
Neuroticism	0.75^*^	−0.05^*^	−0.37^*^	−0.02	−0.05^*^	−0.62^*^	0.05	0.02	0.02	0.00	−0.01
Extraversion	0.73^*^	0.15^*^	−0.10^*^	0.14^*^	0.12^*^	0.22^*^	0.03	0.52^*^	0.14^*^	0.02	−0.10^*^
Openness	0.29^*^	0.00	−0.21^*^	0.14^*^	0.02	0.10^*^	0.49^*^	−0.12^*^	0.03	0.28^*^	−0.36^*^
Agreeableness	0.49^*^	−0.14^*^	0.15^*^	0.17^*^	−0.04	0.19^*^	−0.01	0.08^*^	−0.33^*^	0.50^*^	−0.10^*^
Conscientiousness	0.71^*^	−0.07^*^	0.02	0.62^*^	−0.18^*^	0.19^*^	−0.10^*^	−0.07^*^	0.00	0.04	0.25^*^
**BFI**											
Neuroticism	0.62^*^	−0.05	−0.42^*^	0.07	−0.06^*^	−0.57^*^	0.01	0.02	0.02	0.05	0.05
Extraversion	0.65^*^	0.13^*^	−0.25^*^	0.06	0.08^*^	0.37^*^	−0.04	0.50^*^	0.11^*^	0.02	0.01
Openness	0.33^*^	0.02	−0.16^*^	0.18^*^	0.01	0.12^*^	0.46^*^	−0.13^*^	0.23^*^	0.12^*^	−0.25^*^
Agreeableness	0.54^*^	−0.03	0.28^*^	0.06	−0.02	0.14^*^	−0.10^*^	0.12^*^	−0.15^*^	0.47^*^	−0.03
Conscientiousness	0.70^*^	−0.08^*^	−0.01	0.58^*^	−0.09^*^	0.21^*^	−0.12^*^	−0.04	−0.04	0.03	0.34^*^

N for the relations with the MIDUS and IPIP = 1124–1176. N for the relations with BFI = 1318–1321. Values represent standardized regression coefficients when the models include all 10 PTPI scales as predictors,

* p < 0.01.

#### Predictive validity

As seen in Table 6, the correlations between the PTPI scales and well-being, health, and life satisfaction scales revealed several significant relations (only those above 0.30 are noted below).

**Table 6:** **Correlations between the PTPI scales, well-being, physical health, and life satisfaction**.

**Scale**	**Vigor**	**Calmness**	**Mature personality**	**Impulsiveness**	**Self-confidence**	**Culture**	**Sociability**	**Leadership**	**Social sensitivity**	**Tidiness**
**HBC**										
Wellness maintenance	0.38^*^	0.27^*^	0.34^*^	−0.06	0.25^*^	0.35^*^	0.27^*^	0.24^*^	0.23^*^	0.32^*^
Accident control	0.30^*^	0.35^*^	0.39^*^	−0.04	0.32^*^	0.33^*^	0.30^*^	0.31^*^	0.30^*^	0.34^*^
Traffic risk	0.21^*^	−0.24^*^	−0.20^*^	0.33^*^	−0.07^*^	−0.13^*^	0.03	0.14^*^	−0.27^*^	−0.23^*^
Substance risk	0.03	−0.12^*^	−0.06	0.13^*^	0.00	−0.10^*^	0.03	0.01	−0.06	−0.06
**SF-36**										
Physical Functioning	0.35^*^	0.04	0.07^*^	−0.01	0.09^*^	0.02	0.01	−0.01	−0.05	0.06
Lack physical health limitations	0.28^*^	0.09^*^	0.09^*^	−0.01	0.11^*^	0.05	0.03	0.00	−0.03	0.08^*^
Lack emotional health limitations	0.17^*^	0.18^*^	0.17^*^	−0.04	0.29^*^	0.04	0.09^*^	0.04	0.00	0.12^*^
Energy	0.54^*^	0.30^*^	0.28^*^	0.05	0.44^*^	0.19^*^	0.26^*^	0.21^*^	0.08^*^	0.22^*^
Emotional well being	0.24^*^	0.45^*^	0.33^*^	−0.04	0.58^*^	0.20^*^	0.24^*^	0.14^*^	0.18^*^	0.19^*^
Social Functioning	0.27^*^	0.23^*^	0.20^*^	−0.04	0.33^*^	0.08^*^	0.12^*^	0.01	0.03	0.11^*^
Pain	−0.30^*^	−0.08^*^	−0.04	0.03	−0.13^*^	−0.04	−0.01	0.03	0.06	−0.03
General health	0.47^*^	0.25^*^	0.23^*^	−0.01	0.28^*^	0.19^*^	0.16^*^	0.12^*^	0.12^*^	0.18^*^
**Satisfaction with Life**	0.31^*^	0.31^*^	0.27^*^	0.11^*^	0.38^*^	0.24^*^	0.28^*^	0.26^*^	0.16^*^	0.17^*^

N = 1228–1421.

* p < 0.01.

*Vigor* predicted more wellness maintenance, accident control, physical functioning, energy, general health, life satisfaction, and less pain. *Calmness* predicted more accident control, energy, emotional well-being, and life satisfaction. *Mature personality* predicted more wellness maintenance, accident control, and emotional well-being. *Impulsiveness* predicted more traffic risk. *Self-confidence* predicted more accident control, energy, emotional well-being, social functioning, and life satisfaction. *Culture* predicted more wellness maintenance and accident control. *Sociability*, *leadership*, and *social sensitivity* all predicted more accident control. Finally, *tidiness* predicted more wellness maintenance and accident control.

## Study 2

In this study we investigated the test-retest reliability of the PTPI.

### Methods

#### Participants

For the Wave 1 assessment, we recruited 84 English speaking US participants, in the summer of 2013, via the SocialSci online platform. Two weeks after completing the first assessment, participants were contacted and invited to take the PTPI again (Wave 2). Participants received 50 SocialSci points for completing the first wave and 100 SocialSci points for completing the second wave (300 SocialSci points are equal to a $5 Amazon gift card).

Of the initial 84 people available in Wave 1, 50 retook the test in Wave 2. Prior to any analyses, we excluded 12 participants, because they completed the entire PTPI in less than 5 min, which means they took less than 2 s per question, thus rendering their data of questionable quality. After this exclusion, the final test-retest sample used for the analysis consisted of 38 people. A power analysis showed that this sample size is appropriate, because to detect a moderate effect of 0.5 with a power of 0.8, the required number of participants would be 29.

***Demographics***. Of the remaining 38 participants, there were 20 men and 18 women. Participant ages ranged from 17 to 74 years (*M* = 31.87, *SD* = 13.58) and the sample included a variety of racial backgrounds (33 Caucasian/European Americans, 2 African Americans, 1 Asian American, 1 Hispanic American, and 1 mixed race participant).

***Measure***. As in Study 1, we used the PTPI measure, which includes 150 items, from which we scored the 10 different sub-scales. For each item, participants rated how well the item described them on a 5-point Likert scale ranging from 1 (*Not very well)* to 5 (*Extremely well*).

### Results

All 10 PTPI scales showed very high test-retest reliability, as follows: Vigor (0.91), Calmness (0.89), Mature Personality (0.89), Impulsiveness (0.79), Self-Confidence (0.91), Culture (0.89), Sociability (0.92), Leadership (0.85), Social Sensitivity (0.90), Tidiness (0.93).

When using the original dichotomous coding of the PTPI items in Study 2 (see Measures section), the test-retest reliabilities were as follows: Vigor (0.81), Calmness (0.77), Mature Personality (0.79), Impulsiveness (0.61), Self-Confidence (0.89), Culture (0.82), Sociability (0.80), Leadership (0.76), Social Sensitivity (0.85), Tidiness (0.88). Thus, the average decrement in test-retest reliability was 0.09.

## General discussion

The purpose of the current studies was to establish the internal consistency reliability, construct validity, predictive validity, and test-retest reliability of the PTPI. In Study 1, we examined the construct validity of the PTPI scales by assessing their relations with the MIDUS personality scale, IPIP-NEO, CCS, BFI, Grit-S, and NPI. We examined the predictive validity of the PTPI scales by assessing their relations with physical health and well-being, via the HBC and SF-36, and with life satisfaction via the SWLS. We also investigated differences in PTPI scores across age and gender. In Study 2, we examined the test-retest reliability of the PTPI.

All the PTPI scales showed good internal consistency reliability and test-retest reliability. In terms of defining the content of the PTPI scales, as a whole, the PTPI measures Big Five personality traits fairly well. We used the Big Five as an organizing framework for our test of convergent and discriminant validity for several reasons. First, the Big Five affords an organizing taxonomy of personality traits that can be applied even to inventories that predate the advent of the Big Five. In the early stages of personality research on traits, there was a lack of consensus regarding the appropriate number and content necessary to organize and describe personality. Thus, according to Eysenck (1983) there were three factors, according to Comrey (1970) there were eight factors, according to Cattell et al. (1970) there were 16 factors, and according to Flanagan et al. (1960), who created the PTPI, there were ten. However, in more recent years, personality researchers have finally converged toward a commonly accepted model, namely the Five-Factor Model or the “Big Five.”

The impact and importance of the Five Factor Model can be tracked by looking at the number of publications over time that used this model as opposed to older models. Starting in the late 1990s, the number of Big Five publications greatly overtook the older models. For instance, in 2006, the number of Big Five publications exceeded 300 per year, compared with less than 50 for the two older models (John et al., 2008). Therefore, at this point, the Big Five has been reliably used in thousands of studies, showing the impact of personality on health, achievement, relationships, and a plethora of other important outcomes. This highlights the importance of validation papers such as the present one, where older personality measures are validated against the dominant five-factor model, to help researchers integrate new findings from older but valuable data sets (such as Project Talent) into the context of modern personality research.

We did not, however, find that the PTPI had direct and unique counterparts for each of the Big Five scales and the names of the PTPI scales can be somewhat misleading. For example, the “Social Sensitivity” label could be interpreted as Neuroticism, when in fact the scale correlates most highly with Agreeableness. Below we describe which of the PTPI scales cover best each of the Big Five traits, across construct validation scales. As can be seen in Table 4, the results replicated across the MIDUS, IPIP-NEO, BFI, and CCS.

*Emotional stability* was best captured in the PTPI by the Self-Confidence scale (which was negatively related to Anxiety, Depression, Self-consciousness, and Vulnerability), and to a lesser extent by the Calmness scale (which was negatively related to Anger).

*Extraversion* was best captured in the PTPI by the Sociability scale (which was particularly related to Friendliness, Gregariousness, and Cheerfulness), and to a lesser extent by the Vigor and Self-confidence scales.

*Openness* was best captured in the PTPI by the Culture scale (which was particularly related to Artistic Interests), and to a lesser extent by the Vigor and Leadership scales.

*Agreeableness* was best captured in the PTPI by the Social Sensitivity scale (which was particularly related to Altruism and Sympathy), and to a lesser extent by the Calmness scale.

Finally, *conscientiousness* was best captured in the PTPI by the Mature Personality scale (which was highly related to Self-Efficacy, Dutifulness, Achievement striving, and Self Discipline), and to a lesser extent by the Tidiness scale (which was highly related to Orderliness).

Thus, although the Self-Confidence, Sociability, Culture, Social Sensitivity, and Mature Personality scales may be used as proxies for Emotional Stability, Extraversion, Openness, Agreeableness, and Conscientiousness, respectively, various combinations of PTPI scales should be considered, given the large amount of shared variance among the PTPI scales, and the fact that none of the five PTPI scales fully covers the IPIP-NEO facets of the respective big five trait. For example, the Mature Personality and Tidiness scales could be combined to form a more encompassing proxy for Conscientiousness. Furthermore, researchers should keep in mind that some of the PTPI scales are not very well-captured by any one Big Five trait. For example, Impulsiveness correlates highly with both Extraversion (positively) and with Conscientiousness (negatively), and Leadership correlates highly with Extraversion and Openness (positively) and with Neuroticism (negatively).

The PTPI scales also showed good predictive validity, as they related to physical health, well-being, and life satisfaction outcomes in the expected ways. Thus, Vigor, Calmness, Mature Personality, Culture, and Tidiness were most strongly related to preventive health behaviors such as accident control and wellness maintenance, whereas Impulsiveness was related to risky traffic behaviors. Furthermore, Vigor was most strongly related to self-reported general health, whereas Self-Confidence was most strongly related to self-reported emotional well-being. Self-confidence and Vigor were the scales most strongly related to life satisfaction.

In summary, the present paper brings evidence that the PTPI scales (in their current form) show good reliability and validity, and may be used (albeit with caution) as personality measures of the Big Five, when studying various outcomes as measured in the Project Talent study. With the help of these personality scales, researchers may use the Project Talent data to investigate the prospective role of personality in educational and occupational attainment, health, income, and social status, as well as the prospective role of interactions between personality, interests, socio-economic status, family background, intelligence on achievement and health outcomes.

## Conflict of interest statement

The authors declare that the research was conducted in the absence of any commercial or financial relationships that could be construed as a potential conflict of interest.

## Footnotes

^1^Zoomerang has since been purchased by Survey Monkey and no longer exists as an independent organization.

^2^Note that the Project Talent data file that is available to researchers contains no item-level data of the PTPI (except for a small subsample, 4% of the people, who were not systematically followed up). Thus, researchers interested in using the current Project Talent data and the PTPI scales will have to use the scales as they were originally computed and as reported in this paper (see Appendix and Table A1) It is for this reason that we did not find it necessary to perform an item-level analysis in this paper. That is not to say that an item level analysis would not be of interest to other researchers who might want to use the PTPI in new samples. When, and if that type of research might be conducted, interested researchers will have access to our data and can conduct the item-level factor analysis if they are interested in updating and revising the PTPI scales.

## Appendix

### PTPI instructions

For each statement below mark which the one of the five choices which best describes how the statement applies to you. Regarding the things I do and the way I do them, this statement describes me: 1 (not very well), 2 (slightly), 3 (fairly well), 4 (quite well), 5 (extremely well).

**Table A1:** **PTPI items organized by scale**.

**PTPI Items**	**Coding**
**VIGOR**	
I can work or play outdoors for hours without getting tired.	
I am a fast walker.	
I am full of pep and energy.	
People seem to think I lead a vigorous life.	
I am active.	
I am vigorous.	
I am energetic.	
**CALMNESS**	
I often lose my temper.	R
I can usually keep my wits about me even in difficult situations.	
People seem to think I get angry easily.	R
People seem to think I have good self-control.	
People consider me level-headed.	
I am even-tempered.	
I am calm.	
I am stable.	
I am usually self-controlled.	
**MATURE PERSONALITY**	
I make good use of all my time.	
I never seem to get things done on time.	R
I work fast and get a lot done.	
When I say I'll do something I get it done.	
It bothers me to leave a task half done.	
I can turn out a lot more work than average.	
I am hard-working.	
People consider me an efficient worker.	
I do my job, even when I don't like it.	
I find it hard to keep working toward long-range goals.	R
I am productive.	
As soon as I finish one project or assignment, I always have something else I want to begin.	
I never volunteer for a tough job.	R
I think that if something is worth starting its worth finishing.	
I do things the best I know how, even if no one checks up on me.	
I lose interest in most projects before I get them done.	R
People seem to think they can count on me.	
People consider me persistent.	
I am dependable.	
People have criticized me for leaving things undone.	R
I am conscientious.	
I am persistent.	
I am reliable.	
People consider me determined.	
**IMPULSIVENESS**	
I like to do things on the spur of the moment.	
I usually act on the first plan that comes to mind.	
I feel that I'm impulsive.	
People seem to think I sometimes make decisions too quickly.	
I am impulsive.	
I don't believe in rushing into things.	R
I am cautious.	R
When I have a problem, I make up my mind and don't worry about it.	
It takes me quite a while to come to a decision.	R
**SELF-CONFIDENCE**	
I am confident.	
I'd enjoy speaking to a club group on a subject I know	
Being around strangers makes me ill-at-ease.	R
I'm troubled by people making fun of me.	R
People seem to think my feelings are hurt too easily.	R
I am usually at ease.	
People seem to think I am easily discouraged when criticized.	R
I am often self-conscious.	R
People consider me shy.	R
I am sensitive.	R
I am often worried.	R
People seem to think I usually do a good job on whatever I'm doing.	
**CULTURE**	
I enjoy beautiful things.	
I feel that good manners are very necessary for everyone.	
I think culture is more important than wealth.	
I enjoy cultural things.	
I am a cultured person.	
People seem to think I have good taste.	
I take part in the cultural activities in my community.	
I tend to have good taste.	
I am refined.	
I am sometimes crude.	R
**SOCIABILITY**	
I like to spend a good deal of time by myself.	R
I'd rather be with a group of friends than at home by myself.	
People consider me the quiet type.	R
People seem to think I make new friends more quickly than most people do.	
I couldn't get along without having people around me most of the time.	
I enjoy getting to know people.	
I like to be with people most of the time.	
I go out of my way to be with friends.	
I prefer reading a good book to going out with friends.	R
People consider me good-natured.	
People consider me sociable.	
I am friendly.	
**LEADERSHIP**	
I am the leader in my group.	
I am influential.	
I have held a lot of elected offices.	
People naturally follow my lead.	
I like to make decisions.	
**SOCIAL SENSITIVITY**	
I like to tease people.	R
I never hurt another person's feelings if I can avoid it.	
I seem to know how other people will feel about things.	
I sympathize with my friends and encourage them when they have problems.	
People consider me a sympathetic listener.	
People consider me very helpful in dealing with other people.	
I am sympathetic.	
I am considerate.	
People consider me understanding.	
**TIDINESS**	
I am never sloppy in my personal appearance.	
I have a definite place for all of my things.	
Before I start a task, I spend some time getting it organized.	
It bothers me to be with someone who dresses carelessly.	
I like to do things systematically.	
My work suffers from lack of neatness.	R
People consider me very careful about my personal appearance.	
I am tidy.	
I am neat.	
I am orderly.	
I tend to be untidy.	R

R marks reverse scored items.
